# 5-(Hy­droxy­meth­yl)furan-2-carbaldehyde

**DOI:** 10.1107/S1600536810031119

**Published:** 2010-08-11

**Authors:** Tamila Shalumova, Joseph M. Tanski

**Affiliations:** a Department of Chemistry, Vassar College, Poughkeepsie, NY 12604, USA

## Abstract

The title compound (HMF), C_6_H_6_O_3_, is one of the products of acid-catalyzed dehydration of high-fructose corn syrup, and has been shown to be toxic to honey bees. The compound was crystallized at 276 K, and it was found that the two independent mol­ecules in the asymmetric unit form an infinite O—H⋯O hydrogen-bonding chain that is linked into a three-dimensional network structure by weak inter­molecular C—H⋯O contacts.

## Related literature

For the formation of HMF from high-fructose corn syrup, see: Le Blanc *et al.* (2009 ▶), and the story subsequently reported in *Chemical & Engineering News* by Kemsley (2009 ▶). The effect of HMF on honey bees was studied by Bailey (1966 ▶); for the mechanism of HMF formation from sugars, see: Antal *et al.* (1990 ▶); Haworth & Jones (1944 ▶); Ermolaeva & Sapronova (1982 ▶). For the effect of HMF on DNA, see: Durling *et al.* (2009 ▶).
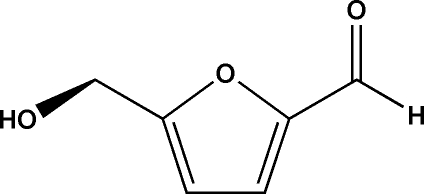


## Experimental

### 

#### Crystal data

C_6_H_6_O_3_
*M* *_r_* = 126.11
Monoclinic, 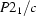

*a* = 15.9126 (17) Å
*b* = 5.6166 (6) Å
*c* = 13.1722 (14) Å
β = 90.770 (2)°
*V* = 1177.2 (2) Å^3^
*Z* = 8
Mo *K*α radiation
μ = 0.12 mm^−1^
*T* = 125 K
0.22 × 0.19 × 0.14 mm

#### Data collection

Bruker APEXII CCD diffractometer
Absorption correction: multi-scan (*SADABS*; Bruker, 2007 ▶) *T*_min_ = 0.975, *T*_max_ = 0.984
15720 measured reflections
2933 independent reflections
2246 reflections with *I* > 2σ(*I*)
*R*_int_ = 0.042

#### Refinement

*R*[*F* ^2^ > 2σ(*F* ^2^)] = 0.036
*wR*(*F* ^2^) = 0.089
*S* = 1.04
2933 reflections
169 parameters
2 restraints
H atoms treated by a mixture of independent and constrained refinement
Δρ_max_ = 0.25 e Å^−3^
Δρ_min_ = −0.21 e Å^−3^

### 

Data collection: *APEX2* (Bruker, 2007 ▶); cell refinement: *SAINT* (Bruker, 2007 ▶); data reduction: *SAINT*; program(s) used to solve structure: *SHELXS97* (Sheldrick, 2008 ▶); program(s) used to refine structure: *SHELXL97* (Sheldrick, 2008 ▶); molecular graphics: *SHELXTL* (Sheldrick, 2008 ▶); software used to prepare material for publication: *SHELXL97*.

## Supplementary Material

Crystal structure: contains datablocks I, global. DOI: 10.1107/S1600536810031119/si2283sup1.cif

Structure factors: contains datablocks I. DOI: 10.1107/S1600536810031119/si2283Isup2.hkl

Additional supplementary materials:  crystallographic information; 3D view; checkCIF report

## Figures and Tables

**Table 1:** Hydrogen-bond geometry (Å, °)

*D*—H⋯*A*	*D*—H	H⋯*A*	*D*⋯*A*	*D*—H⋯*A*
O13—H13*O*⋯O23^i^	0.85 (1)	1.89 (1)	2.7341 (13)	175 (2)
O23—H23*O*⋯O11^ii^	0.84 (1)	1.87 (1)	2.7006 (14)	173 (2)
C14—H14*A*⋯O13^iii^	0.95	2.41	3.3029 (17)	156
C21—H21*A*⋯O21^iv^	0.95	2.56	3.4726 (15)	160
C23—H23*B*⋯O21^iii^	0.95	2.38	3.3258 (16)	175
C24—H24*A*⋯O23^iii^	0.95	2.46	3.3734 (16)	160
C26—H26*A*⋯O21^v^	0.99	2.53	3.4639 (16)	158

Symmetry codes: (i) 
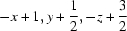
; (ii) 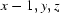; (iii) 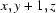; (iv) 
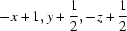
; (v) 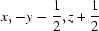.

**Fig. 1.**
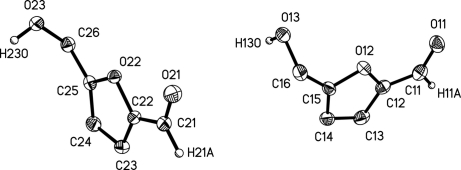
A view of the two independent moleucles of HMF, with displacement ellipsoids shown at the 50% probability level. H atoms on carbon, except for the H atoms on the aldehydes, have been omitted for clarity.

**Fig. 2.**
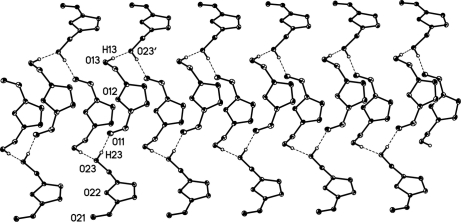
A view of the one-dimensional hydrogen bonding chain formed by the two independent moleucles of HMF. H atoms on carbon have been omitted for clarity.

## supplementary crystallographic information

### Comment

5-(Hydroxymethyl)-2-furancarboxaldehyde (Scheme 1), or, as it is more commonly referred to as 5-hydroxymethylfurfural, HMF, is formed by acid-catalyzed dehydration of sugars, most notably of D-fructose (Antal *et al.*, 1990; Bailey, 1966; Ermolaeva & Sapronova, 1982; Haworth & Jones, 1944). It is present in many foods such as dried fruit, coffee, and bread, and especially in food that has been heated (Durling *et al.*, 2009). HMF is also formed by acid-catalyzed degradation of high-fructose corn syrup that has been subject to heat. It is toxic to honey bees, which are fed high-fructose corn syrup by beekeepers to promote colony growth and when nectar sources are scarce (Kemsley, 2009; Le Blanc *et al.*, 2009). The toxicity presents itself to bees as intestinal ulcerations, which lead to dysentery and, soon after, death. One study by Durling *et al.*, (2009) has shown that HMF may damage DNA.

The asymmetric unit contains two independent unique molecules of HMF (Figure 1) which are hydrogen bonded into an infinite one-dimensional screw-like chain along the crystallographic *b* axis (Figure 2, Table 1). The hydroxymethyl oxygen O23 is both a hydrogen bond donor and acceptor. The aldehyde oxygen of one of the independent molecules, O11, acts as a hydrogen bond acceptor from the proton on O23 of the second independent molecule, *D*···*A* 2.701 (1) Å. The proton on the hydroxylmethyl oxygen of the first independent molecule, O13, acts as a hydrogen bond donor to the hydroxymethyl oxygen O23, *D*···*A* 2.734 (1) Å. The aldehyde oxygen of the second molecule, O21, is not involved in classical hydrogen bonding, however it is involved in C—H···O interactions. Five weak intermolecular C—H···O contacts (Table 1) link the screw-like hydrogen bonded chains into a three-dimensional network structure.

### Experimental

5-Hydroxymethylfurfural was purchased from Aldrich and used without further purification. The compound was placed in a 276 K cold room until crystallization occurred. A crystal suitable for diffraction was selected and mounted in a nylon loop with Paratone-*N* cryoprotectant oil with a microscope in the cold room before being placed immediately in a 125 K coldstream on the diffractometer.

### Refinement

A suitable crystal was mounted in a nylon loop with Paratone-*N* cryoprotectant oil and data was collected on a Bruker *APEXII* CCD platform diffractometer. The structure was solved using direct methods and standard difference map techniques, and was refined by full-matrix least-squares procedures on *F^2^* with *SHELXTL* Version 6.14 (Sheldrick, 2008). All non-hydrogen atoms were refined anisotropically. Hydrogen atoms on carbon were included in calculated positions with distances C—H = 0.95 - 0.99 Å and were refined using a riding model, with *U*_iso_(H) = 1.2*U*_eq_(C). Hydrogen atoms on oxygen were refined semifreely with the help of a distance restraint d(O–H) = 0.84 Å, and *U*_iso_(H) = 1.2*U*_eq_(O).The extinction parameter (EXTI) refined to zero and was removed from the refinement.

### Crystal data

**Table:**

C_6_H_6_O_3_	*F*(000) = 528
*M**_r_* = 126.11	*D*_x_ = 1.423 Mg m^−^^3^
Monoclinic, *P*2_1_/*c*	Melting point = 301–307 K
Hall symbol: -P 2ybc	Mo *K*α radiation, λ = 0.71073 Å
*a* = 15.9126 (17) Å	Cell parameters from 5970 reflections
*b* = 5.6166 (6) Å	θ = 2.6–28.2°
*c* = 13.1722 (14) Å	µ = 0.12 mm^−^^1^
β = 90.770 (2)°	*T* = 125 K
*V* = 1177.2 (2) Å^3^	Block, colourless
*Z* = 8	0.22 × 0.19 × 0.14 mm

### Data collection

**Table:**

Bruker APEXII CCD diffractometer	2933 independent reflections
Radiation source: fine-focus sealed tube	2246 reflections with *I* > 2σ(*I*)
graphite	*R*_int_ = 0.042
φ and ω scans	θ_max_ = 28.3°, θ_min_ = 2.6°
Absorption correction: multi-scan (*SADABS*; Bruker, 2007)	*h* = −21→21
*T*_min_ = 0.975, *T*_max_ = 0.984	*k* = −7→7
15720 measured reflections	*l* = −17→17

### Refinement

**Table:**

Refinement on *F*^2^	Primary atom site location: structure-invariant direct methods
Least-squares matrix: full	Secondary atom site location: difference Fourier map
*R*[*F*^2^ > 2σ(*F*^2^)] = 0.036	Hydrogen site location: inferred from neighbouring sites
*wR*(*F*^2^) = 0.089	H atoms treated by a mixture of independent and constrained refinement
*S* = 1.04	*w* = 1/[σ^2^(*F*_o_^2^) + (0.0367*P*)^2^ + 0.3118*P*] where *P* = (*F*_o_^2^ + 2*F*_c_^2^)/3
2933 reflections	(Δ/σ)_max_ = 0.001
169 parameters	Δρ_max_ = 0.25 e Å^−^^3^
2 restraints	Δρ_min_ = −0.21 e Å^−^^3^

### Special details

**Table:**

Geometry. All e.s.d.'s (except the e.s.d. in the dihedral angle between two l.s. planes) are estimated using the full covariance matrix. The cell e.s.d.'s are taken into account individually in the estimation of e.s.d.'s in distances, angles and torsion angles; correlations between e.s.d.'s in cell parameters are only used when they are defined by crystal symmetry. An approximate (isotropic) treatment of cell e.s.d.'s is used for estimating e.s.d.'s involving l.s. planes.
Refinement. Refinement of *F*^2^ against ALL reflections. The weighted *R*-factor *wR* and goodness of fit *S* are based on *F*^2^, conventional *R*-factors *R* are based on *F*, with *F* set to zero for negative *F*^2^. The threshold expression of *F*^2^ > σ(*F*^2^) is used only for calculating *R*-factors(gt) *etc*. and is not relevant to the choice of reflections for refinement. *R*-factors based on *F*^2^ are statistically about twice as large as those based on *F*, and *R*- factors based on ALL data will be even larger.

### Fractional atomic coordinates and isotropic or equivalent isotropic displacement parameters (Å^2^)

**Table:**

	*x*	*y*	*z*	*U*_iso_*/*U*_eq_	
O11	1.11183 (6)	0.07149 (19)	0.62286 (8)	0.0320 (2)	
O12	0.94229 (6)	0.19067 (16)	0.61025 (7)	0.0227 (2)	
O13	0.77247 (6)	0.02624 (17)	0.68104 (8)	0.0268 (2)	
H13O	0.7607 (10)	0.125 (3)	0.7278 (11)	0.032*	
O21	0.44841 (6)	−0.14117 (17)	0.33477 (7)	0.0259 (2)	
O22	0.38382 (5)	0.01084 (15)	0.52416 (6)	0.01771 (19)	
O23	0.25895 (6)	−0.14054 (16)	0.67299 (7)	0.0229 (2)	
H23O	0.2152 (9)	−0.064 (3)	0.6587 (12)	0.027*	
C11	1.08966 (9)	0.2796 (3)	0.63013 (10)	0.0278 (3)	
H11A	1.1321	0.3966	0.6401	0.033*	
C12	1.00405 (9)	0.3586 (2)	0.62456 (10)	0.0246 (3)	
C13	0.96973 (10)	0.5808 (3)	0.62951 (10)	0.0295 (3)	
H13B	0.9989	0.7269	0.6386	0.035*	
C14	0.88169 (10)	0.5496 (3)	0.61829 (10)	0.0290 (3)	
H14A	0.8402	0.6713	0.6189	0.035*	
C15	0.86787 (8)	0.3121 (2)	0.60650 (10)	0.0230 (3)	
C16	0.79019 (9)	0.1669 (3)	0.59398 (10)	0.0271 (3)	
H16A	0.7421	0.2743	0.5801	0.032*	
H16B	0.7964	0.0608	0.5345	0.032*	
C21	0.44388 (8)	0.0692 (2)	0.35668 (10)	0.0209 (3)	
H21A	0.4642	0.1803	0.3085	0.025*	
C22	0.41029 (7)	0.1641 (2)	0.44951 (9)	0.0187 (3)	
C23	0.39749 (8)	0.3945 (2)	0.47805 (10)	0.0220 (3)	
H23B	0.4111	0.5334	0.4405	0.026*	
C24	0.35976 (8)	0.3853 (2)	0.57519 (10)	0.0235 (3)	
H24A	0.3423	0.5172	0.6149	0.028*	
C25	0.35344 (8)	0.1518 (2)	0.60018 (9)	0.0186 (3)	
C26	0.32435 (8)	0.0284 (2)	0.69336 (10)	0.0215 (3)	
H26A	0.3726	−0.0546	0.7257	0.026*	
H26B	0.3037	0.1486	0.7420	0.026*	

### Atomic displacement parameters (Å^2^)

**Table:**

	*U*^11^	*U*^22^	*U*^33^	*U*^12^	*U*^13^	*U*^23^
O11	0.0247 (5)	0.0384 (6)	0.0327 (6)	0.0042 (4)	−0.0022 (4)	0.0009 (5)
O12	0.0223 (5)	0.0214 (5)	0.0244 (5)	0.0033 (4)	−0.0016 (4)	−0.0015 (4)
O13	0.0294 (5)	0.0216 (5)	0.0294 (5)	0.0024 (4)	0.0051 (4)	−0.0041 (4)
O21	0.0299 (5)	0.0234 (5)	0.0244 (5)	0.0013 (4)	0.0036 (4)	−0.0020 (4)
O22	0.0193 (4)	0.0157 (4)	0.0182 (4)	0.0001 (3)	0.0023 (3)	0.0005 (3)
O23	0.0216 (5)	0.0214 (5)	0.0258 (5)	0.0011 (4)	0.0041 (4)	0.0035 (4)
C11	0.0283 (7)	0.0353 (8)	0.0198 (7)	−0.0048 (6)	−0.0006 (5)	−0.0007 (6)
C12	0.0294 (7)	0.0252 (7)	0.0191 (6)	−0.0038 (5)	0.0012 (5)	−0.0004 (5)
C13	0.0409 (8)	0.0235 (7)	0.0244 (7)	−0.0023 (6)	0.0079 (6)	−0.0018 (6)
C14	0.0377 (8)	0.0245 (7)	0.0251 (7)	0.0083 (6)	0.0092 (6)	0.0022 (6)
C15	0.0264 (7)	0.0251 (7)	0.0176 (6)	0.0081 (5)	0.0022 (5)	0.0011 (5)
C16	0.0254 (7)	0.0320 (8)	0.0238 (7)	0.0056 (6)	−0.0001 (5)	−0.0014 (6)
C21	0.0186 (6)	0.0233 (7)	0.0210 (6)	0.0006 (5)	0.0012 (5)	0.0037 (5)
C22	0.0162 (6)	0.0192 (6)	0.0206 (6)	−0.0014 (5)	0.0007 (5)	0.0039 (5)
C23	0.0221 (6)	0.0175 (6)	0.0263 (7)	0.0003 (5)	0.0008 (5)	0.0029 (5)
C24	0.0241 (7)	0.0193 (6)	0.0273 (7)	0.0024 (5)	0.0024 (5)	−0.0026 (5)
C25	0.0164 (6)	0.0194 (6)	0.0200 (6)	0.0019 (5)	0.0003 (5)	−0.0020 (5)
C26	0.0215 (6)	0.0233 (6)	0.0197 (6)	0.0011 (5)	0.0013 (5)	−0.0014 (5)

### Geometric parameters (Å, °)

**Table:**

O11—C11	1.2249 (18)	C14—C15	1.360 (2)
O12—C15	1.3670 (15)	C14—H14A	0.9500
O12—C12	1.3732 (16)	C15—C16	1.488 (2)
O13—C16	1.4239 (17)	C16—H16A	0.9900
O13—H13O	0.850 (13)	C16—H16B	0.9900
O21—C21	1.2188 (16)	C21—C22	1.4431 (17)
O22—C25	1.3698 (14)	C21—H21A	0.9500
O22—C22	1.3769 (14)	C22—C23	1.3639 (18)
O23—C26	1.4312 (16)	C23—C24	1.4217 (19)
O23—H23O	0.838 (13)	C23—H23B	0.9500
C11—C12	1.434 (2)	C24—C25	1.3561 (18)
C11—H11A	0.9500	C24—H24A	0.9500
C12—C13	1.364 (2)	C25—C26	1.4888 (18)
C13—C14	1.418 (2)	C26—H26A	0.9900
C13—H13B	0.9500	C26—H26B	0.9900
			
C15—O12—C12	106.27 (10)	C15—C16—H16B	109.0
C16—O13—H13O	105.8 (11)	H16A—C16—H16B	107.8
C25—O22—C22	105.96 (9)	O21—C21—C22	125.62 (12)
C26—O23—H23O	107.5 (11)	O21—C21—H21A	117.2
O11—C11—C12	124.46 (13)	C22—C21—H21A	117.2
O11—C11—H11A	117.8	C23—C22—O22	110.37 (11)
C12—C11—H11A	117.8	C23—C22—C21	129.96 (12)
C13—C12—O12	110.41 (12)	O22—C22—C21	119.65 (11)
C13—C12—C11	131.41 (14)	C22—C23—C24	106.27 (11)
O12—C12—C11	118.17 (12)	C22—C23—H23B	126.9
C12—C13—C14	106.13 (13)	C24—C23—H23B	126.9
C12—C13—H13B	126.9	C25—C24—C23	106.68 (11)
C14—C13—H13B	126.9	C25—C24—H24A	126.7
C15—C14—C13	106.91 (13)	C23—C24—H24A	126.7
C15—C14—H14A	126.5	C24—C25—O22	110.71 (11)
C13—C14—H14A	126.5	C24—C25—C26	132.47 (12)
C14—C15—O12	110.28 (12)	O22—C25—C26	116.75 (11)
C14—C15—C16	133.06 (13)	O23—C26—C25	112.81 (10)
O12—C15—C16	116.63 (11)	O23—C26—H26A	109.0
O13—C16—C15	112.84 (11)	C25—C26—H26A	109.0
O13—C16—H16A	109.0	O23—C26—H26B	109.0
C15—C16—H16A	109.0	C25—C26—H26B	109.0
O13—C16—H16B	109.0	H26A—C26—H26B	107.8

### Hydrogen-bond geometry (Å, °)

**Table:**

*D*—H···*A*	*D*—H	H···*A*	*D*···*A*	*D*—H···*A*
O13—H13O···O23^i^	0.85 (1)	1.89 (1)	2.7341 (13)	175.(2)
O23—H23O···O11^ii^	0.84 (1)	1.87 (1)	2.7006 (14)	173.(2)
C14—H14A···O13^iii^	0.95	2.41	3.3029 (17)	156.
C21—H21A···O21^iv^	0.95	2.56	3.4726 (15)	160.
C23—H23B···O21^iii^	0.95	2.38	3.3258 (16)	175.
C24—H24A···O23^iii^	0.95	2.46	3.3734 (16)	160.
C26—H26A···O21^v^	0.99	2.53	3.4639 (16)	158.

Symmetry codes: (i) −*x*+1, *y*+1/2, −*z*+3/2; (ii) *x*−1, *y*, *z*; (iii) *x*, *y*+1, *z*; (iv) −*x*+1, *y*+1/2, −*z*+1/2; (v) *x*, −*y*−1/2, *z*+1/2.
